# Supplementary material for: Development of a preoperative index-based nomogram for the prediction of hypokalemia in patients with pituitary adenoma: a retrospective cohort study
Source: PeerJ. 2021 Jul 19;9:e11650. doi: 10.7717/peerj.11650 (PMC8297473; doi:10.7717/peerj.11650)
Supplement: Supplemental Information 4 [file peerj-09-11650-s004.docx]

| Supplement Table 2 Patients with missing data in each of the variables | | |
| --- | --- | --- |
| Variables | Number of missing | Percentage of missing |
| age | 1 | 0.004878049 |
| sex | 0 | 0 |
| education | 22 | 0.107317073 |
| marriage | 1 | 0.004878049 |
| smoking | 1 | 0.004878049 |
| driking | 1 | 0.004878049 |
| SBP | 4 | 0.019512195 |
| DBP | 4 | 0.019512195 |
| hypertension | 3 | 0.014634146 |
| history of hypertension | 1 | 0.004878049 |
| heart rate | 5 | 0.024390244 |
| diabetes | 2 | 0.009756098 |
| type of pituitary adenomas | 2 | 0.009756098 |
| type of pituitary adenomas 2 | 2 | 0.009756098 |
| Preoperative medication | 9 | 0.043902439 |
| Preoperative surgical treatment | 9 | 0.043902439 |
| Preoperative radiotherapy | 9 | 0.043902439 |
| tumor diameter | 18 | 0.087804878 |
| weight | 30 | 0.146341463 |
| height | 28 | 0.136585366 |
| bmi | 32 | 0.156097561 |
| pt | 2 | 0.009756098 |
| pta | 1 | 0.004878049 |
| ptr | 1 | 0.004878049 |
| ptnir | 1 | 0.004878049 |
| fbg | 2 | 0.009756098 |
| aptt | 1 | 0.004878049 |
| tt | 1 | 0.004878049 |
| dd | 3 | 0.014634146 |
| alt | 3 | 0.014634146 |
| ast | 8 | 0.03902439 |
| Total bilirubin | 4 | 0.019512195 |
| Direct bilirubin | 11 | 0.053658537 |
| Indirect bilirubin | 15 | 0.073170732 |
| ggt | 7 | 0.034146341 |
| alp | 3 | 0.014634146 |
| k | 2 | 0.009756098 |
| na | 5 | 0.024390244 |
| cl | 2 | 0.009756098 |
| ga | 4 | 0.019512195 |
| p | 11 | 0.053658537 |
| mg | 11 | 0.053658537 |
| cysc | 20 | 0.097560976 |
| Urea | 6 | 0.029268293 |
| Creac | 3 | 0.014634146 |
| Uric Acid Nitrogen and Creatinine | 142 | 0.692682927 |
| Carbon dioxide binding capacity | 2 | 0.009756098 |
| ua | 2 | 0.009756098 |
| glu | 1 | 0.004878049 |
| Hydroxybutyric acid | 20 | 0.097560976 |
| chol | 11 | 0.053658537 |
| tg | 12 | 0.058536585 |
| hdlc | 13 | 0.063414634 |
| ldlc | 11 | 0.053658537 |
| Apolipoprotein a | 12 | 0.058536585 |
| Apolipoprotein b | 12 | 0.058536585 |
| Apolipoprotein e | 64 | 0.312195122 |
| pa | 12 | 0.058536585 |
| tp | 5 | 0.024390244 |
| ALB | 4 | 0.019512195 |
| glb | 5 | 0.024390244 |
| Albumin globulin | 11 | 0.053658537 |
| tba | 17 | 0.082926829 |
| cg | 129 | 0.629268293 |
| ck | 13 | 0.063414634 |
| ldh | 9 | 0.043902439 |
| ckmb | 11 | 0.053658537 |
| c-reactive protein | 20 | 0.097560976 |
| ceh | 5 | 0.024390244 |
| lap | 45 | 0.219512195 |
| rbp | 66 | 0.32195122 |
| afu | 12 | 0.058536585 |
| lip | 15 | 0.073170732 |
| samy | 13 | 0.063414634 |
| Fe | 13 | 0.063414634 |
| uibc | 11 | 0.053658537 |
| tibc | 11 | 0.053658537 |
| Iron saturation | 145 | 0.707317073 |
| sf | 128 | 0.624390244 |
| tf | 12 | 0.058536585 |
| ada | 15 | 0.073170732 |
| sod | 12 | 0.058536585 |
| nefa | 13 | 0.063414634 |
| wbc | 3 | 0.014634146 |
| rbc | 2 | 0.009756098 |
| hgb | 1 | 0.004878049 |
| plt | 1 | 0.004878049 |
| hct | 2 | 0.009756098 |
| mcv | 4 | 0.019512195 |
| mch | 3 | 0.014634146 |
| mchc | 2 | 0.009756098 |
| rdwcv | 17 | 0.082926829 |
| rdwsd | 3 | 0.014634146 |
| lym | 1 | 0.004878049 |
| neut | 1 | 0.004878049 |
| mono | 2 | 0.009756098 |
| eos | 6 | 0.029268293 |
| baso | 1 | 0.004878049 |
| lyw | 1 | 0.004878049 |
| neutnum | 1 | 0.004878049 |
| mononum | 2 | 0.009756098 |
| eosnum | 1 | 0.004878049 |
| basonum | 2 | 0.009756098 |
| pct | 4 | 0.019512195 |
| mpv | 7 | 0.034146341 |
| pdw | 6 | 0.029268293 |
| plcr | 5 | 0.024390244 |
| ret | 3 | 0.014634146 |
| retnum | 3 | 0.014634146 |
| irf | 3 | 0.014634146 |
| hypokalemia | 0 | 0 |
