# Supplementary material for: Development of a preoperative index-based nomogram for the prediction of hypokalemia in patients with pituitary adenoma: a retrospective cohort study
Source: PeerJ. 2021 Jul 19;9:e11650. doi: 10.7717/peerj.11650 (PMC8297473; doi:10.7717/peerj.11650)
Supplement: Supplemental Information 5 [file peerj-09-11650-s005.docx]

| Supplement Table 3 Difference of original datasets and the complete dataset after multiple imputations | | | | |
| --- | --- | --- | --- | --- |
| Variables | Total (n = 410) | 1 (n = 205) | 2 (n = 205) | p |
| Age, Mean ± SD | 47.33 ± 14.15 | 47.35 ± 14.18 | 47.30 ± 14.16 | 0.974 |
| Gender, n (%) |  |  |  |  |
| Male | 208 (51) | 104 (51) | 104 (51) | 1 |
| Female | 202 (49) | 101 (49) | 101 (49) |  |
| Education, n (%) |  |  |  |  |
| Junior high school and below | 238 (65) | 107 (66) | 131 (64) | 0.69 |
| High school and above | 128 (35) | 54 (34) | 74 (36) |  |
| States of marriage, n (%) |  |  |  |  |
| Marriage | 353 (86) | 176 (86) | 177 (86) | 1 |
| Others | 56 (14) | 28 (14) | 28 (14) |  |
| Smoking, n (%) |  |  |  |  |
| Yes | 42 (10) | 21 (10) | 21 (10) | 1 |
| No | 367 (90) | 183 (90) | 184 (90) |  |
| Drinking, n (%) |  |  |  |  |
| Yes | 8 ( 2) | 4 ( 2) | 4 ( 2) | 1 |
| No | 401 (98) | 200 (98) | 201 (98) |  |
| SBP, Mean ± SD | 80.67 ± 12.94 | 80.64 ± 12.98 | 80.70 ± 12.93 | 0.959 |
| DBP, Mean ± SD | 124.92 ± 20.10 | 124.91 ± 20.16 | 124.93 ± 20.08 | 0.99 |
| Hypertension, n (%) |  |  |  |  |
| No | 269 (66) | 133 (66) | 136 (66) | 0.999 |
| Yes | 138 (34) | 69 (34) | 69 (34) |  |
| History of hypertension, n (%) |  |  |  |  |
| Yes | 88 (22) | 44 (22) | 44 (21) | 1 |
| No | 321 (78) | 160 (78) | 161 (79) |  |
| Heart rate, Mean ± SD | 79.40 ± 13.20 | 79.42 ± 13.19 | 79.38 ± 13.24 | 0.973 |
| Diabetes, n (%) |  |  |  |  |
| No | 331 (81) | 165 (81) | 166 (81) | 1 |
| Yes | 77 (19) | 38 (19) | 39 (19) |  |
| Type of pituitary, n (%) |  |  |  |  |
| Nonfunctioning pituitary adenomas | 324 (79) | 161 (79) | 163 (80) | 1 |
| ACTH- pituitary adenomas | 24 ( 6) | 12 ( 6) | 12 ( 6) |  |
| PRL- pituitary adenomas | 20 ( 5) | 10 ( 5) | 10 ( 5) |  |
| GH- pituitary adenomas | 34 ( 8) | 17 ( 8) | 17 ( 8) |  |
| Others | 6 ( 1) | 3 ( 1) | 3 ( 1) |  |
| Type of pituitary2, n (%) |  |  |  |  |
| Nonfunctioning pituitary adenomas | 324 (79) | 161 (79) | 163 (80) | 1 |
| Functioning pituitary adenomas | 84 (21) | 42 (21) | 42 (20) |  |
| Preoperative medication, n (%) |  |  |  |  |
| Yes | 106 (26) | 51 (26) | 55 (27) | 0.944 |
| No | 295 (74) | 145 (74) | 150 (73) |  |
| Preoperative surgical, n (%) |  |  |  |  |
| Yes | 57 (14) | 28 (14) | 29 (14) | 1 |
| No | 344 (86) | 168 (86) | 176 (86) |  |
| Preoperative radiotherapy, n (%) |  |  |  |  |
| Yes | 14 ( 3) | 6 ( 3) | 8 ( 4) | 0.852 |
| No | 387 (97) | 190 (97) | 197 (96) |  |
| Tumor diameter, Mean ± SD | 2.59 ± 1.40 | 2.61 ± 1.39 | 2.58 ± 1.41 | 0.828 |
| Weight, Mean ± SD | 65.20 ± 11.20 | 65.15 ± 11.27 | 65.24 ± 11.17 | 0.944 |
| Height, Mean ± SD | 161.55 ± 11.99 | 161.20 ± 12.70 | 161.74 ± 11.60 | 0.706 |
| BMI, Mean ± SD | 25.44 ± 4.85 | 25.19 ± 4.73 | 25.58 ± 4.92 | 0.484 |
| PT, Mean ± SD | 11.48 ± 0.90 | 11.48 ± 0.90 | 11.48 ± 0.90 | 0.985 |
| PTA, Mean ± SD | 100.84 ± 19.68 | 100.75 ± 19.65 | 100.92 ± 19.75 | 0.932 |
| PTR, Mean ± SD | 1.00 ± 0.08 | 1.00 ± 0.08 | 1.00 ± 0.08 | 0.971 |
| PTNIR, Mean ± SD | 1.00 ± 0.08 | 1.00 ± 0.08 | 1.00 ± 0.08 | 0.996 |
| Fbg, Mean ± SD | 3.12 ± 0.93 | 3.12 ± 0.93 | 3.12 ± 0.92 | 0.976 |
| APTT, Mean ± SD | 26.93 ± 5.07 | 26.90 ± 5.04 | 26.96 ± 5.12 | 0.894 |
| TT, Mean ± SD | 18.43 ± 1.43 | 18.43 ± 1.43 | 18.42 ± 1.44 | 0.939 |
| DD, Median (IQR) | 0.24 (0.15, 0.40) | 0.24 (0.15, 0.40) | 0.24 (0.15, 0.40) | 0.883 |
| Alt, Median (IQR) | 22.00 (17.00, 30.00) | 22.00 (17.00, 29.00) | 22.00 (17.00, 30.00) | 0.816 |
| Ast, Median (IQR) | 20.50 (15.00, 31.00) | 20.00 (15.00, 31.00) | 21.00 (15.00, 31.00) | 0.641 |
| Total bilirubin, Median (IQR) | 11.70 (9.10, 15.25) | 11.70 (9.10, 15.30) | 11.70 (9.10, 15.10) | 0.969 |
| Direct bilirubin, Median (IQR) | 1.90 (1.50, 2.70) | 1.90 (1.50, 2.70) | 1.90 (1.50, 2.70) | 0.905 |
| Indirect bilirubin, Mean ± SD | 10.38 ± 3.86 | 10.31 ± 3.79 | 10.44 ± 3.93 | 0.735 |
| GGT, Median (IQR) | 26.00 (17.00, 40.00) | 25.50 (17.00, 39.50) | 26.00 (17.00, 41.00) | 0.735 |
| ALP, Mean ± SD | 79.57 ± 23.69 | 79.73 ± 23.69 | 79.40 ± 23.74 | 0.888 |
| K, Mean ± SD | 3.99 ± 0.31 | 3.99 ± 0.31 | 3.99 ± 0.31 | 0.976 |
| Na, Mean ± SD | 139.54 ± 3.56 | 139.62 ± 3.40 | 139.46 ± 3.71 | 0.661 |
| CL, Median (IQR) | 105.0 (103.38, 106.60) | 105.0 (103.35, 106.5) | 105.0 (103.40, 106.60) | 0.935 |
| Ga, Mean ± SD | 2.31 ± 0.11 | 2.31 ± 0.11 | 2.31 ± 0.11 | 0.949 |
| P, Median (IQR) | 1.20 (1.05, 1.37) | 1.20 (1.05, 1.37) | 1.20 (1.05, 1.37) | 0.963 |
| Mg, Mean ± SD | 0.87 ± 0.08 | 0.87 ± 0.08 | 0.87 ± 0.08 | 0.785 |
| Cysc, Mean ± SD | 0.82 ± 0.20 | 0.81 ± 0.20 | 0.82 ± 0.20 | 0.774 |
| Urea, Mean ± SD | 4.69 ± 1.35 | 4.67 ± 1.33 | 4.71 ± 1.37 | 0.778 |
| Creac, Mean ± SD | 73.95 ± 18.20 | 73.87 ± 18.20 | 74.03 ± 18.24 | 0.928 |
| Carbon dioxide binding capacity, | 24.85 ± 3.18 | 24.84 ± 3.17 | 24.85 ± 3.20 | 0.959 |
| UA, Mean ± SD | 24.84 ± 3.15 | 24.84 ± 3.15 | 24.84 ± 3.15 | 1 |
| Glucose, Mean ± SD | 5.43 ± 1.94 | 5.41 ± 1.93 | 5.44 ± 1.96 | 0.887 |
| Hydroxybutyric acid, Median (IQR) | 0.08 (0.07, 0.11) | 0.08 (0.07, 0.10) | 0.08 (0.07, 0.11) | 0.409 |
| TC, Mean ± SD | 5.27 ± 1.44 | 5.27 ± 1.44 | 5.28 ± 1.45 | 0.916 |
| TG, Median (IQR) | 1.69 (1.08, 2.67) | 1.64 (1.07, 2.59) | 1.69 (1.11, 2.67) | 0.705 |
| HDL-C, Mean ± SD | 1.17 ± 0.29 | 1.16 ± 0.29 | 1.17 ± 0.29 | 0.736 |
| LDL-C, Mean ± SD | 3.42 ± 1.02 | 3.41 ± 1.02 | 3.42 ± 1.03 | 0.918 |
| Apolipoproteina, Mean ± SD | 1.20 ± 0.25 | 1.20 ± 0.25 | 1.21 ± 0.25 | 0.717 |
| Apolipoproteinb, Mean ± SD | 1.01 ± 0.30 | 1.01 ± 0.29 | 1.02 ± 0.30 | 0.73 |
| PA, Mean ± SD | 0.29 ± 0.10 | 0.29 ± 0.09 | 0.29 ± 0.10 | 0.786 |
| TP, Mean ± SD | 70.07 ± 6.22 | 70.09 ± 6.23 | 70.05 ± 6.23 | 0.937 |
| ALB, Mean ± SD | 40.75 ± 4.08 | 40.72 ± 4.06 | 40.77 ± 4.10 | 0.902 |
| Glb, Mean ± SD | 29.25 ± 4.37 | 29.27 ± 4.31 | 29.23 ± 4.44 | 0.924 |
| Albumin globulin, Mean ± SD | 1.42 ± 0.26 | 1.42 ± 0.26 | 1.42 ± 0.27 | 0.931 |
| Tba, Median (IQR) | 3.10 (2.10, 5.10) | 3.10 (2.10, 5.03) | 3.10 (2.10, 5.20) | 0.774 |
| CK, Median (IQR) | 97.00 (66.00, 135.00) | 97.00 (64.75, 134.25) | 97.00 (66.00, 136.00) | 0.815 |
| LDH, Mean ± SD | 205.27 ± 71.26 | 204.69 ± 71.40 | 205.82 ± 71.30 | 0.874 |
| CKMB, Mean ± SD | 13.28 ± 5.06 | 13.16 ± 4.98 | 13.39 ± 5.15 | 0.664 |
| C-reactive protein, Median (IQR) | 1.25 (0.45, 3.20) | 1.23 (0.40, 3.01) | 1.31 (0.47, 3.50) | 0.357 |
| CEH, Mean ± SD | 8037.63 ± 2059.99 | 8038.05 ± 2064.03 | 8037.22 ± 2061.10 | 0.997 |
| AFU, Mean ± SD | 27.51 ± 8.74 | 27.28 ± 8.57 | 27.72 ± 8.92 | 0.618 |
| LIP, Median (IQR) | 35.00 (27.00, 49.00) | 35.00 (27.00, 48.00) | 35.00 (27.00, 50.00) | 0.658 |
| SAMY, Mean ± SD | 71.13 ± 25.62 | 70.61 ± 25.23 | 71.62 ± 26.03 | 0.696 |
| Fe, Mean ± SD | 16.45 ± 6.34 | 16.31 ± 6.19 | 16.57 ± 6.49 | 0.687 |
| Uibc, Mean ± SD | 38.47 ± 13.25 | 38.30 ± 12.97 | 38.63 ± 13.55 | 0.802 |
| Tibc, Mean ± SD | 54.71 ± 13.04 | 54.56 ± 12.50 | 54.86 ± 13.56 | 0.818 |
| TF, Mean ± SD | 2.37 ± 0.65 | 2.36 ± 0.63 | 2.38 ± 0.67 | 0.727 |
| ADA, Mean ± SD | 10.49 ± 3.42 | 10.45 ± 3.40 | 10.53 ± 3.45 | 0.807 |
| SOD, Mean ± SD | 148.54 ± 18.66 | 149.15 ± 18.15 | 147.97 ± 19.16 | 0.527 |
| Nefa, Mean ± SD | 447.24 ± 224.46 | 444.30 ± 221.14 | 450.00 ± 228.03 | 0.801 |
| WBC, Mean ± SD | 7.00 ± 2.48 | 7.00 ± 2.47 | 7.00 ± 2.51 | 0.993 |
| RBC, Mean ± SD | 4.57 ± 0.60 | 4.58 ± 0.60 | 4.57 ± 0.61 | 0.969 |
| HGB, Mean ± SD | 131.04 ± 18.59 | 130.97 ± 18.58 | 131.11 ± 18.65 | 0.939 |
| PLT, Mean ± SD | 258.78 ± 65.42 | 258.86 ± 65.56 | 258.70 ± 65.44 | 0.98 |
| HCT, Mean ± SD | 0.40 ± 0.05 | 0.40 ± 0.05 | 0.40 ± 0.05 | 0.971 |
| MCV, Median (IQR) | 88.10 (85.00, 91.50) | 88.10 (85.00, 91.50) | 88.10 (85.00, 91.50) | 0.997 |
| MCH, Median (IQR) | 29.60 (28.30, 30.50) | 29.60 (28.35, 30.50) | 29.60 (28.30, 30.50) | 0.956 |
| MCHC, Mean ± SD | 331.31 ± 14.51 | 331.39 ± 14.37 | 331.23 ± 14.69 | 0.909 |
| RDMCH, n (%) |  |  |  |  |
| 0.11 | 4 ( 1) | 2 ( 1) | 2 ( 1) | 0.998 |
| 0.12 | 88 (22) | 42 (22) | 46 (22) |  |
| 0.13 | 179 (46) | 85 (45) | 94 (46) |  |
| 0.14 | 90 (23) | 43 (23) | 47 (23) |  |
| 0.15 | 32 ( 8) | 16 ( 9) | 16 ( 8) |  |
| RDWSD, Mean ± SD | 42.16 ± 4.32 | 42.13 ± 4.26 | 42.20 ± 4.39 | 0.869 |
| Percentage Lym, Mean ± SD | 32.06 ± 11.78 | 32.09 ± 11.80 | 32.04 ± 11.79 | 0.968 |
| Percentage Neut, Mean ± SD | 58.71 ± 13.56 | 58.70 ± 13.59 | 58.71 ± 13.56 | 0.992 |
| Percentage MONO, Mean ± SD | 6.24 ± 2.38 | 6.22 ± 2.36 | 6.25 ± 2.39 | 0.904 |
| Percentage EOS, Median (IQR) | 1.85 (1.00, 3.30) | 1.80 (1.00, 3.10) | 1.90 (1.00, 3.50) | 0.619 |
| Percentage BASO, Median (IQR) | 0.30 (0.10, 0.50) | 0.30 (0.10, 0.50) | 0.30 (0.10, 0.50) | 0.982 |
| LYW, Mean ± SD | 2.06 ± 0.73 | 2.07 ± 0.73 | 2.06 ± 0.73 | 0.942 |
| Neut, Mean ± SD | 4.35 ± 2.45 | 4.36 ± 2.45 | 4.35 ± 2.45 | 0.968 |
| MONO, Mean ± SD | 0.43 ± 0.23 | 0.43 ± 0.23 | 0.43 ± 0.23 | 0.999 |
| EOS, Mean ± SD | 0.16 ± 0.16 | 0.16 ± 0.16 | 0.16 ± 0.16 | 0.892 |
| BASO, Mean ± SD | 0.02 ± 0.02 | 0.02 ± 0.02 | 0.02 ± 0.02 | 0.926 |
| PCT, Mean ± SD | 0.27 ± 0.07 | 0.27 ± 0.07 | 0.27 ± 0.07 | 0.862 |
| MPV, Mean ± SD | 10.45 ± 1.03 | 10.45 ± 1.02 | 10.45 ± 1.04 | 0.991 |
| PDW Median (IQR) | 11.65 (10.60, 13.12) | 11.60 (10.60, 13.10) | 11.70 (10.60, 13.20) | 0.921 |
| PLCR, Mean ± SD | 28.43 ± 8.77 | 28.42 ± 8.77 | 28.43 ± 8.80 | 0.994 |
| Percentage RET, Median (IQR) | 1.41 (1.10, 1.75) | 1.41 (1.11, 1.76) | 1.40 (1.10, 1.74) | 0.847 |
| RET, Mean ± SD | 66.83 ± 26.53 | 66.52 ± 26.21 | 67.13 ± 26.90 | 0.817 |
| IRF, Median (IQR) | 7.10 (4.90, 10.90) | 7.10 (4.90, 10.90) | 7.10 (4.90, 10.90) | 0.985 |
| Postoperative hypokalemia, n (%) |  |  |  |  |
| No | 254 (62) | 127 (62) | 127 (62) | 1 |
| Yes | 156 (38) | 78 (38) | 78 (38) |  |
