# Supplementary material for: Development of a preoperative index-based nomogram for the prediction of hypokalemia in patients with pituitary adenoma: a retrospective cohort study
Source: PeerJ. 2021 Jul 19;9:e11650. doi: 10.7717/peerj.11650 (PMC8297473; doi:10.7717/peerj.11650)
Supplement: Supplemental Information 6 [file peerj-09-11650-s006.docx]

| Supplement Table 4 Comparison of clinical and biochemical parameters between the training and validation sets | | | | |
| --- | --- | --- | --- | --- |
| Variables | Total (n = 205) | Training set (n = 143) | validation set (n = 62) | P |
| Age, Mean ± SD | 47.30 ± 14.16 | 47.38 ± 14.13 | 47.11 ± 14.35 | 0.901 |
| Sex, n (%) |  |  |  | 0.772 |
| Male | 104 (51) | 74 (52) | 30 (48) |  |
| Female | 101 (49) | 69 (48) | 32 (52) |  |
| Education, n (%) |  |  |  | 0.362 |
| Junior high school and below | 131 (64) | 88 (62) | 43 (69) |  |
| High school and above | 74 (36) | 55 (38) | 19 (31) |  |
| States of marriage, n (%) |  |  |  | 0.648 |
| Marriage | 177 (86) | 125 (87) | 52 (84) |  |
| Others | 28 (14) | 18 (13) | 10 (16) |  |
| Smoking, n (%) |  |  |  | 0.353 |
| Yes | 21 (10) | 17 (12) | 4 ( 6) |  |
| No | 184 (90) | 126 (88) | 58 (94) |  |
| Srinking, n (%) |  |  |  | 1 |
| Yes | 4 ( 2) | 3 ( 2) | 1 ( 2) |  |
| No | 201 (98) | 140 (98) | 61 (98) |  |
| SBP, Mean ± SD | 80.70 ± 12.93 | 79.89 ± 12.98 | 82.58 ± 12.73 | 0.169 |
| DBP, Mean ± SD | 124.93± 20.08 | 124.69 ± 21.03 | 125.50 ± 17.85 | 0.777 |
| Hypertension, n (%) |  |  |  | 0.136 |
| No | 136 (66) | 100 (70) | 36 (58) |  |
| Yes | 69 (34) | 43 (30) | 26 (42) |  |
| History of hypertension, n (%) | |  |  | 0.659 |
| Yes | 44 (21) | 29 (20) | 15 (24) |  |
| No | 161 (79) | 114 (80) | 47 (76) |  |
| Heart rate, Mean ± SD | 79.38 ± 13.24 | 80.24 ± 14.06 | 77.40 ± 10.95 | 0.122 |
| Diabetes, n (%) |  |  |  | 0.374 |
| No | 166 (81) | 113 (79) | 53 (85) |  |
| Yes | 39 (19) | 30 (21) | 9 (15) |  |
| Type of pituitary adenomas, n (%) |  |  |  | 0.912 |
| Nonfunctioning | 163 (80) | 115 (80) | 48 (77) |  |
| ACTH- pituitary adenomas | 12 ( 6) | 7 ( 5) | 5 ( 8) |  |
| PRL- pituitary adenomas | 10 ( 5) | 7 ( 5) | 3 ( 5) |  |
| GH- pituitary adenomas | 17 ( 8) | 12 ( 8) | 5 ( 8) |  |
| Others functioning | 3 ( 1) | 2 ( 1) | 1 ( 2) |  |
| Type of pituitary adenomas, n (%) | |  |  | 0.764 |
| Nonfunctioning | 163 (80) | 115 (80) | 48 (77) |  |
| Functioning pituitary adenomas | 42 (20) | 28 (20) | 14 (23) |  |
| Preoperative medication, n (%) | |  |  | 0.522 |
| Yes | 55 (27) | 36 (25) | 19 (31) |  |
| No | 150 (73) | 107 (75) | 43 (69) |  |
| Preoperative surgical treatment, n (%) | |  |  | 0.579 |
| Yes | 29 (14) | 22 (15) | 7 (11) |  |
| No | 176 (86) | 121 (85) | 55 (89) |  |
| Preoperative radiotherapy, n (%) | |  |  | 1 |
| Yes | 8 ( 4) | 6 ( 4) | 2 ( 3) |  |
| No | 197 (96) | 137 (96) | 60 (97) |  |
| Tumor diameter, Mean ± SD | 2.58 ± 1.41 | 2.53 ± 1.38 | 2.69 ± 1.49 | 0.474 |
| Weight, Mean ± SD | 65.24 ± 11.17 | 65.03 ± 10.31 | 65.72 ± 13.04 | 0.713 |
| Height, Mean ± SD | 161.74± 11.60 | 161.99 ± 10.05 | 161.18 ± 14.65 | 0.692 |
| BMI, Mean ± SD | 25.58 ± 4.92 | 25.68 ± 5.13 | 25.37 ± 4.44 | 0.663 |
| PT, Mean ± SD | 11.48 ± 0.90 | 11.46 ± 0.93 | 11.52 ± 0.85 | 0.666 |
| PTA, Mean ± SD | 100.92± 19.75 | 101.58 ± 20.31 | 99.41 ± 18.48 | 0.457 |
| PTR, Mean ± SD | 1.00 ± 0.08 | 1.00 ± 0.08 | 1.00 ± 0.07 | 0.625 |
| PTNIR, Mean ± SD | 1.00 ± 0.08 | 1.00 ± 0.08 | 1.00 ± 0.08 | 0.669 |
| Fbg, Mean ± SD | 3.12 ± 0.92 | 3.17 ± 1.00 | 3.03 ± 0.73 | 0.285 |
| APTT, Mean ± SD | 26.96 ± 5.12 | 27.02 ± 5.07 | 26.82 ± 5.28 | 0.801 |
| TT, Mean ± SD | 18.42 ± 1.44 | 18.41 ± 1.50 | 18.45 ± 1.30 | 0.823 |
| DD, Median (IQR) | 0.24(0.15,0.40) | 0.24 (0.15, 0.42) | 0.24 (0.18, 0.39) | 0.856 |
| Alt, Median (IQR) | 22.00(17.00,30.00) | 23.00 (16.50, 30.50) | 22.00 (18.00, 29.50) | 0.953 |
| Ast, Median (IQR) | 21.00 (15.00, 31.00) | 20.00 (15.00, 32.00) | 21.00 (16.00, 30.00) | 0.708 |
| Total bilirubin, Median (IQR) | 11.70 (9.10, 15.10) | 11.90 (9.10, 14.65) | 11.30 (9.12, 17.78) | 0.483 |
| Direct bilirubin, Median (IQR) | 1.90 (1.50, 2.70) | 1.90 (1.50, 2.65) | 1.85 (1.30, 3.10) | 0.652 |
| Indirect bilirubin, Mean ± SD | 10.44 ± 3.93 | 10.24 ± 3.53 | 10.92 ± 4.73 | 0.309 |
| GGT, Median (IQR) | 26.00 (17.00, 41.00) | 25.00 (16.50, 39.00) | 26.00 (19.25, 44.00) | 0.494 |
| ALP, Mean ± SD | 79.40 ± 23.74 | 80.02 ± 24.34 | 77.97 ± 22.43 | 0.559 |
| K, Mean ± SD | 3.99 ± 0.31 | 3.97 ± 0.32 | 4.03 ± 0.27 | 0.124 |
| Na, Mean ± SD | 139.46 ± 3.71 | 139.47 ± 3.87 | 139.44 ± 3.35 | 0.951 |
| CL, Median (IQR) | 105.00(103.40,106.60) | 105.10(102.95,106.60) | 105.00(103.82,106.40) | 0.441 |
| Ga, Mean ± SD | 2.31 ± 0.11 | 2.31 ± 0.12 | 2.32 ± 0.10 | 0.479 |
| P, Median (IQR) | 1.20 (1.05, 1.37) | 1.20 (1.04, 1.37) | 1.22 (1.08, 1.34) | 0.693 |
| Mg, Mean ± SD | 0.87 ± 0.08 | 0.87 ± 0.08 | 0.86 ± 0.09 | 0.518 |
| Cysc, Mean ± SD | 0.82 ± 0.20 | 0.82 ± 0.21 | 0.81 ± 0.17 | 0.667 |
| Urea, Mean ± SD | 4.71 ± 1.37 | 4.79 ± 1.43 | 4.53 ± 1.21 | 0.176 |
| Creac, Mean ± SD | 74.03 ± 18.24 | 74.98 ± 17.76 | 71.85 ± 19.25 | 0.277 |
| Carbon dioxide binding capacity, | 24.85 ± 3.20 | 24.84 ± 3.28 | 24.89 ± 3.06 | 0.92 |
| UA, Mean ± SD | 24.84 ± 3.15 | 24.82 ± 3.20 | 24.89 ± 3.06 | 0.88 |
| Glucose, Mean ± SD | 5.44 ± 1.96 | 5.50 ± 2.20 | 5.31 ± 1.27 | 0.451 |
| Hydroxybutyric acid, Median (IQR) | 0.08 (0.07, 0.11) | 0.08 (0.06, 0.11) | 0.08 (0.07, 0.11) | 0.49 |
| TC, Mean ± SD | 5.28 ± 1.45 | 5.29 ± 1.46 | 5.25 ± 1.43 | 0.864 |
| TG, Median (IQR) | 1.69 (1.11, 2.67) | 1.74 (1.15, 2.69) | 1.58 (1.02, 2.56) | 0.296 |
| HDL-C, Mean ± SD | 1.17 ± 0.29 | 1.18 ± 0.31 | 1.15 ± 0.26 | 0.414 |
| LDL-C, Mean ± SD | 3.42 ± 1.03 | 3.42 ± 1.02 | 3.43 ± 1.06 | 0.911 |
| Apolipoproteina, Mean ± SD | 1.21 ± 0.25 | 1.21 ± 0.26 | 1.21 ± 0.24 | 0.932 |
| Apolipoproteinb, Mean ± SD | 1.02 ± 0.30 | 1.02 ± 0.30 | 1.02 ± 0.31 | 0.967 |
| PA, Mean ± SD | 0.29 ± 0.10 | 0.29 ± 0.10 | 0.29 ± 0.10 | 0.668 |
| TP, Mean ± SD | 70.05 ± 6.23 | 69.79 ± 6.52 | 70.63 ± 5.51 | 0.348 |
| ALB, Mean ± SD | 40.77 ± 4.10 | 40.61 ± 4.11 | 41.16 ± 4.08 | 0.378 |
| Glb, Mean ± SD | 29.23 ± 4.44 | 29.15 ± 4.55 | 29.42 ± 4.19 | 0.679 |
| Albumin globulin, Mean ± SD | 1.42 ± 0.27 | 1.42 ± 0.26 | 1.43 ± 0.29 | 0.736 |
| Tba, Median (IQR) | 3.10 (2.10, 5.20) | 2.90 (2.00, 5.35) | 3.50 (2.52, 4.75) | 0.446 |
| CK, Median (IQR) | 97.00 (66.00, 136.00) | 94.00 (66.50, 134.50) | 101.50 (65.50, 136.75) | 0.887 |
| LDH, Mean ± SD | 205.82 ± 71.30 | 210.33 ± 76.04 | 195.42 ± 58.17 | 0.128 |
| CKMB, Mean ± SD | 13.39 ± 5.15 | 13.83 ± 5.39 | 12.35 ± 4.43 | 0.042 |
| C-reactive protein, Median (IQR) | 1.31 (0.47, 3.50) | 1.36 (0.52, 5.51) | 0.92 (0.38, 2.86) | 0.178 |
| CEH, Mean ± SD | 8037.22 ± 2061.10 | 7888.53 ± 2062.52 | 8380.18 ± 2032.93 | 0.116 |
| AFU, Mean ± SD | 27.72 ± 8.92 | 27.56 ± 9.27 | 28.08 ± 8.12 | 0.687 |
| LIP, Median (IQR) | 35.00 (27.00, 50.00) | 34.00 (27.00, 51.50) | 36.00 (29.00, 49.00) | 0.754 |
| SAMY, Mean ± SD | 71.62 ± 26.03 | 73.41 ± 27.48 | 67.49 ± 21.99 | 0.104 |
| Fe, Mean ± SD | 16.57 ± 6.49 | 16.31 ± 6.08 | 17.18 ± 7.38 | 0.418 |
| Uibc, Mean ± SD | 38.63 ± 13.55 | 38.79 ± 13.89 | 38.28 ± 12.83 | 0.8 |
| Tibc, Mean ± SD | 54.86 ± 13.56 | 54.59 ± 13.84 | 55.49 ± 12.97 | 0.654 |
| TF, Mean ± SD | 2.38 ± 0.67 | 2.34 ± 0.70 | 2.46 ± 0.59 | 0.195 |
| ADA, Mean ± SD | 10.53 ± 3.45 | 10.61 ± 3.42 | 10.35 ± 3.55 | 0.625 |
| SOD, Mean ± SD | 147.97 ± 19.16 | 146.69 ± 20.15 | 150.92 ± 16.42 | 0.117 |
| Nefa, Mean ± SD | 450.00 ± 228.03 | 454.24 ± 245.88 | 440.21 ± 181.68 | 0.651 |
| WBC, Mean ± SD | 7.00 ± 2.51 | 7.02 ± 2.51 | 6.96 ± 2.52 | 0.873 |
| RBC, Mean ± SD | 4.57 ± 0.61 | 4.56 ± 0.61 | 4.60 ± 0.60 | 0.689 |
| HGB, Mean ± SD | 131.11 ± 18.65 | 131.12 ± 18.90 | 131.08 ± 18.20 | 0.988 |
| PLT, Mean ± SD | 258.70 ± 65.44 | 255.73 ± 64.65 | 265.55 ± 67.27 | 0.333 |
| HCT, Mean ± SD | 0.40 ± 0.05 | 0.40 ± 0.05 | 0.40 ± 0.05 | 0.993 |
| MCV, Median (IQR) | 88.10 (85.00, 91.50) | 88.50 (85.10, 91.80) | 87.05 (84.53, 91.15) | 0.253 |
| MCH, Median (IQR) | 29.60 (28.30, 30.50) | 29.60 (28.50, 30.50) | 29.45 (27.80, 30.55) | 0.56 |
| MCHC, Mean ± SD | 331.23 ± 14.69 | 331.71 ± 13.44 | 330.13 ± 17.31 | 0.524 |
| RDMCH, n (%) |  |  |  | 0.727 |
| 0.11 | 2 ( 1) | 1 ( 1) | 1 ( 2) |  |
| 0.12 | 46 (22) | 31 (22) | 15 (24) |  |
| 0.13 | 94 (46) | 69 (48) | 25 (40) |  |
| 0.14 | 47 (23) | 32 (22) | 15 (24) |  |
| 0.15 | 16 ( 8) | 10 ( 7) | 6 (10) |  |
| RDWSD, Mean ± SD | 42.20 ± 4.39 | 42.20 ± 4.27 | 42.18 ± 4.70 | 0.967 |
| Percentage Lym, Mean ± SD | 32.04 ± 11.79 | 31.76 ± 11.54 | 32.70 ± 12.42 | 0.611 |
| Percentage Neut, Mean ± SD | 58.71 ± 13.56 | 58.94 ± 13.33 | 58.19 ± 14.18 | 0.723 |
| Percentage MONO, Mean ± SD | 6.25 ± 2.39 | 6.22 ± 2.57 | 6.33 ± 1.95 | 0.727 |
| Percentage EOS, Median (IQR) | 1.90 (1.00, 3.50) | 2.00 (1.05, 3.60) | 1.85 (0.95, 2.68) | 0.616 |
| Percentage BASO, Median (IQR) | 0.30 (0.10, 0.50) | 0.30 (0.10, 0.50) | 0.30 (0.10, 0.50) | 0.846 |
| LYW, Mean ± SD | 2.06 ± 0.73 | 2.06 ± 0.71 | 2.07 ± 0.77 | 0.939 |
| Neut, Mean ± SD | 4.35 ± 2.45 | 4.37 ± 2.41 | 4.29 ± 2.56 | 0.832 |
| MONO, Mean ± SD | 0.43 ± 0.23 | 0.44 ± 0.24 | 0.43 ± 0.18 | 0.81 |
| EOS, Mean ± SD | 0.16 ± 0.16 | 0.17 ± 0.16 | 0.15 ± 0.15 | 0.396 |
| BASO, Mean ± SD | 0.02 ± 0.02 | 0.02 ± 0.02 | 0.02 ± 0.02 | 0.831 |
| PCT, Mean ± SD | 0.27 ± 0.07 | 0.26 ± 0.06 | 0.28 ± 0.07 | 0.08 |
| MPV, Mean ± SD | 10.45 ± 1.04 | 10.39 ± 0.99 | 10.57 ± 1.16 | 0.3 |
| PDW Median (IQR) | 11.70 (10.60, 13.20) | 11.50 (10.60, 13.10) | 11.80 (10.60, 13.30) | 0.614 |
| PLCR, Mean ± SD | 28.43 ± 8.80 | 27.99 ± 8.47 | 29.45 ± 9.50 | 0.299 |
| Percentage RET, Median (IQR) | 1.40 (1.10, 1.74) | 1.37 (1.15, 1.73) | 1.44 (1.09, 1.78) | 0.489 |
| RET, Mean ± SD | 67.13 ± 26.90 | 65.82 ± 26.54 | 70.14 ± 27.69 | 0.302 |
| IRF, Median (IQR) | 7.10 (4.90, 10.90) | 7.00 (4.85, 11.20) | 7.20 (5.03, 10.07) | 0.897 |
| Postoperative hypokalemia, n (%) |  |  |  | 1 |
| No | 127 (62) | 89 (62) | 38 (61) |  |
| Yes | 78 (38) | 54 (38) | 24 (39) |  |
