# Supplementary material for: Development of a preoperative index-based nomogram for the prediction of hypokalemia in patients with pituitary adenoma: a retrospective cohort study
Source: PeerJ. 2021 Jul 19;9:e11650. doi: 10.7717/peerj.11650 (PMC8297473; doi:10.7717/peerj.11650)
Supplement: Supplemental Information 7 [file peerj-09-11650-s007.docx]

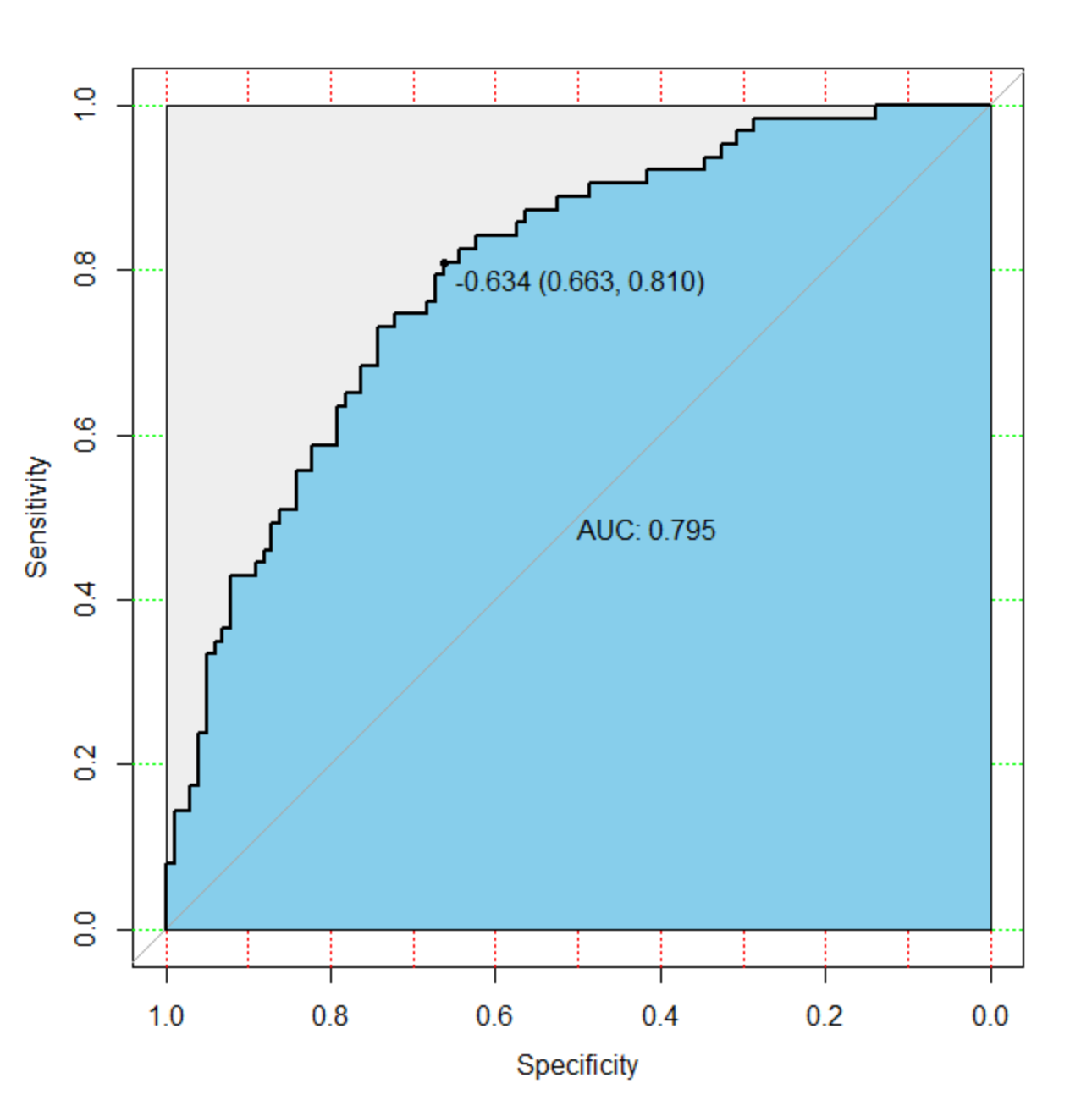


***Figure 8.2 Receiver operating characteristic curves (ROC) for the preoperative index’s prediction model predicting postoperative hypokalemia in sensitivity analysis dataset. AUC, area under the curve.***


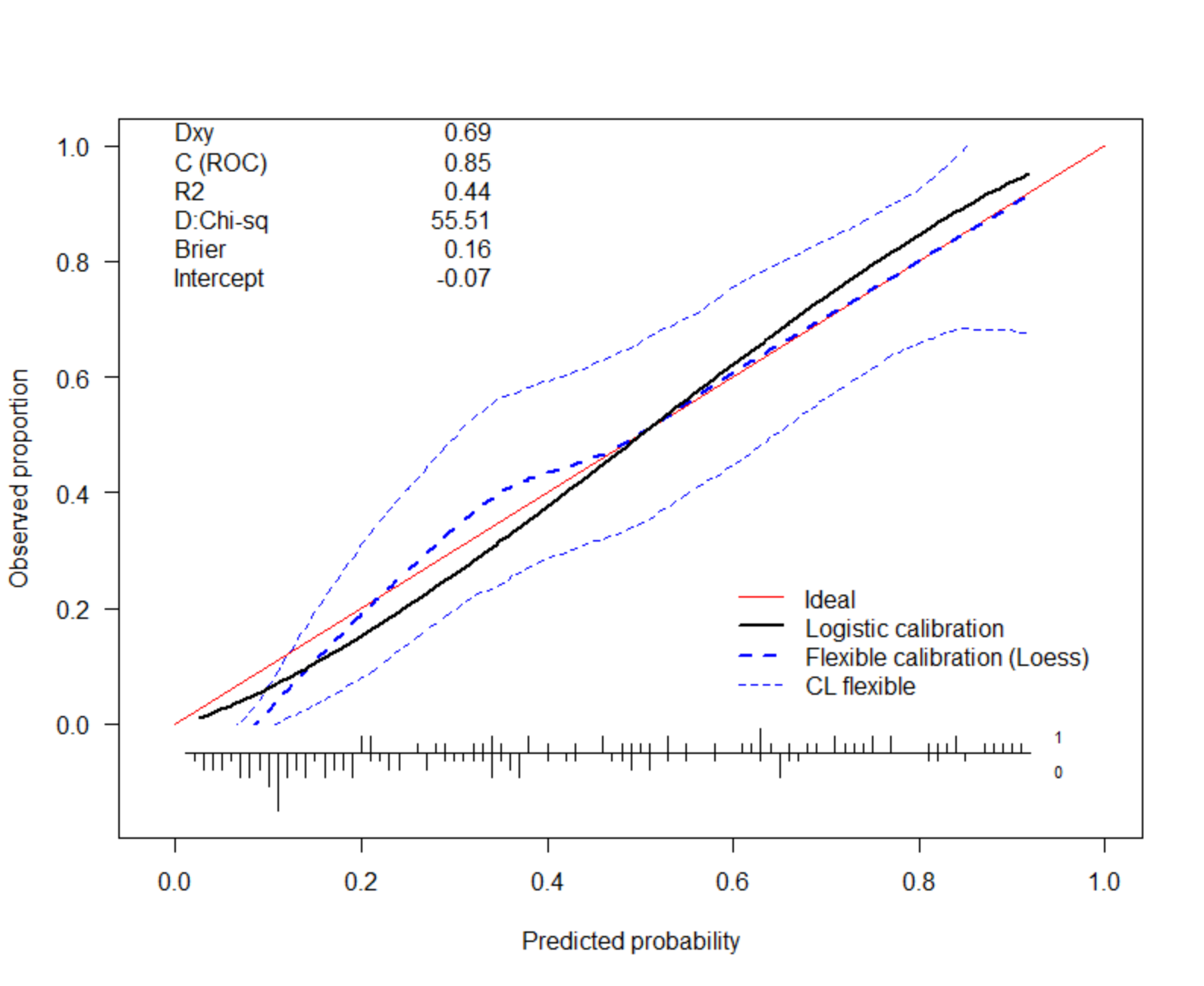


***Figure 8.3 Calibration curve of the preoperative indices’ nomogram. The y-axis represents the actual postoperative hypokalemia rate. The x-axis represents the predicted postoperative hypokalemia risk. The diagonal dotted represents a perfect prediction by an ideal model. The dotted line represents the performance of the nomogram, of which a closer fit to the diagonal dotted line represents a better prediction.***
